# High Circular Polarized Nanolaser with Chiral Gammadion Metal Cavity

**DOI:** 10.1038/s41598-020-64836-1

**Published:** 2020-05-12

**Authors:** Cheng-Li Yu, Yu-Hao Hsiao, Chiao-Yun Chang, Pi-Ju Cheng, Hsiang-Ting Lin, Ming-Sheng Lai, Hao-Chung Kuo, Shu-Wei Chang, Min-Hsiung Shih

**Affiliations:** 1 Research Center for Applied Sciences, Academia Sinica, Taipei, 11529 Taiwan; 2 Department of Photonics and Institute of Electro-Optical Engineering, National Chiao Tung University, Hsinchu, 30010 Taiwan; 3 Department of Photonics, National Sun Yat-sen University, Kaohsiung, 80424 Taiwan

**Keywords:** Lasers, LEDs and light sources, Nanophotonics and plasmonics

## Abstract

We demonstrate a circularly polarized laser with the metal-gallium-nitride gammadion nanocavities. The ultraviolet lasing signal was observed with the high circular dichroism at room temperature under pulsed optical pump conditions. Without external magnetism which breaks the time-reversal symmetry to favor optical transitions of a chosen handedness, the coherent outputs of these chiral nanolasers show a dissymmetry factor as high as 1.1. The small footprint of these lasers are advantageous for applications related to circularly polarized photons in future integrated systems, in contrast to the bulky setup of linearly-polarized lasers and quarter-wave plates.

## Introduction

Compact and coherent circularly-polarized (CP) photon source is a critical element for a wide range of applications such as optical communication^1^, quantum optical information processing^2^, biomedical diagnosis^3^, and display systems^4^. However, the conventional scheme for creating CP waves requires external linear polarizers and quarter-wave plates, which are bulky setups in terms of optical wavelengths. With the external magnetism, it is possible to break the time-reversal (TR) symmetry of light sources so that optical transitions corresponding to a given handedness are selected for CP photon emission^5–9^. However, the corresponding degree of circular polarization is limited by the Curie temperature of magnetic materials^10^ and spin relaxation time of carriers in gain media^11,12^. The long-term compatibilities between magnetism and optoelectronic devices also await further examinations. It is challenging to realize chip-scale semiconductor CP light sources^13–20^, especially, lasers^21–25^. The demonstration of nanoscale semiconductor lasers which directly output simulated emitted photons in a designated CP state at room temperature is in fact nontrivial.

Surface plasmons of metallic structures are effective means to the subwavelength field confinement at optical frequencies. In addition to the dielectric-based structures and cavities^26–30^, metal-dielectric nanostructures are commonly employed in recent research of nanocavities and nanolasers^31–42^. The field confinement and enhancement due to surface plasmons of metal cavities effectively break the diffraction limit. In this work, utilizing gammadion metal cavities and gallium nitride (GaN) at ultraviolet (UV) wavelengths, we demonstrated chiral semiconductor nanolasers with a high degree of circular polarization at room temperature. Their magnitudes of dissymmetry factors are as high as 1.1, indicating that about 77.5% of photons in a specific CP state are output from the nanocavity. We note that the degree of circular polarization here reflects the inherent characteristics of chiral resonant modes inside the metal-GaN gammadion cavities rather than the passive filtering effect of a generic chiral meta-surface.

## Results and Discussion

**Figure 1:**
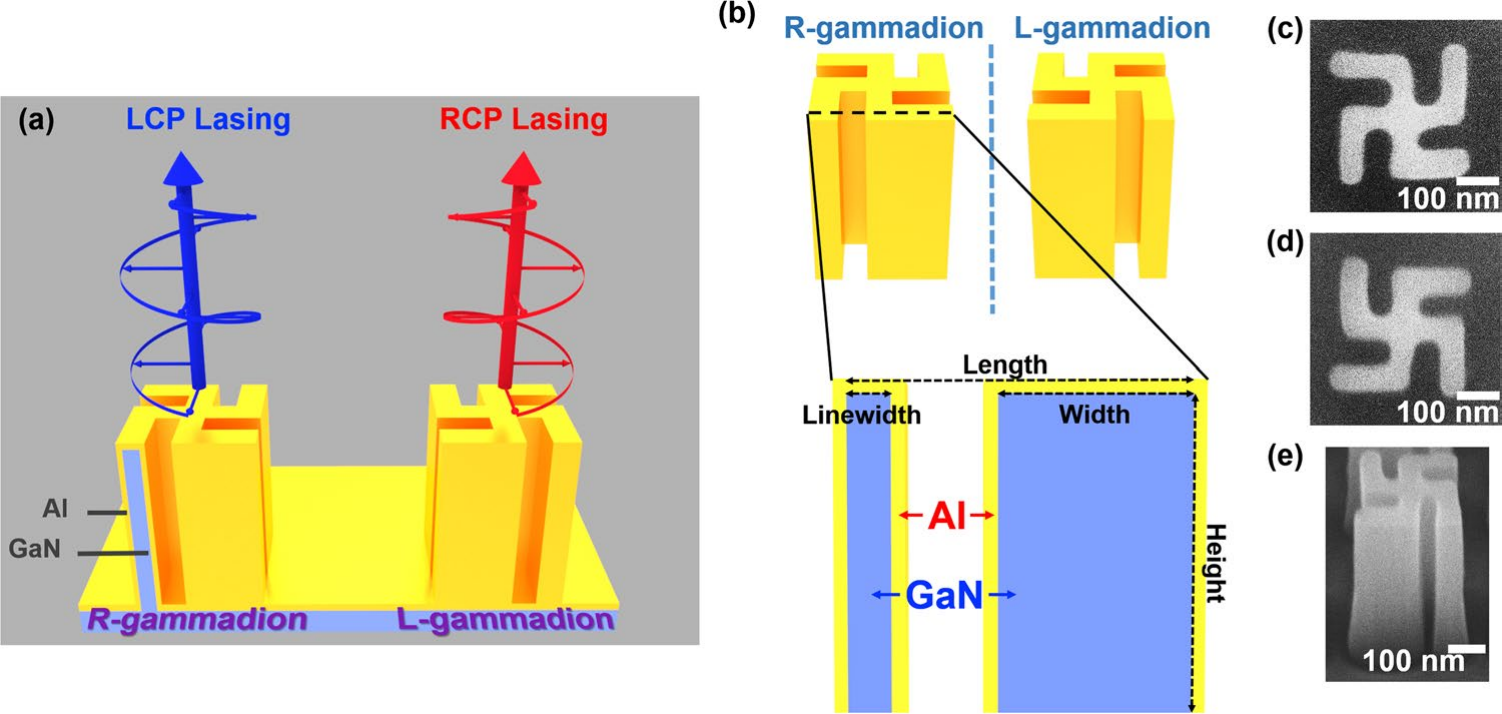
The structures of gammadion metal cavities. (**a**) Schematic diagrams of the metal-GaN R- and L-gammadions. (**b**) The vertical cross section for an R-gammadion cavity. The SEM images (top view) of an (**c**) R-gammadion metal cavity and (**d**) L-counterpart, and (**e**) side view of an L-gammadion in experiment.

The gammadion cavities are shown in Fig. 1a. The right-hand (R-) gammadion is defined as the chiral structure whose arms bend counter-clockwise from center to outer ends, and the one whose arms bend in the opposite manner is the left-hand (L-) counterpart. As indicated in Fig. 1b, the linewidth, width, arm length, and height of GaN gammadions are 50, 300, 500, and 500 nm, respectively. The gammadions were coated with 50 nm thick aluminum (Al) for UV plasmon resonances (see Methods). The scanning-electron-microscope (SEM) images for the two gammadions (top view) are shown in Fig. 1c,d, respectively, and the cross section of an L-gammadion (side view) are illustrated in Fig. 1e. Ideal gammadions have the 4-fold rotation symmetry around the growth direction of GaN (*z* direction), and related symmetry operations belong to the group ${C}_{4}$. We can classify their cavity modes according to how the modal field ${\bf{E}}({\bf{r}})$ transforms into itself under the rotation of $\pi /2$ around the *z* axis ${{\bf{R}}}_{\pi /2}$:

1 $${{\bf{R}}}_{\pi /2}{\bf{E}}({{\bf{R}}}_{\pi /2}^{-1}{\bf{r}})=\chi {\bf{E}}({\bf{r}})$$

where $\chi ={i}^{n}$ ($n=0$ to 3) is the character of the rotation in the four irreducible representations of group ${C}_{4}$. Modes with $\chi =i$and $-i$ may couple to the left-hand CP (LCP) and right-hand CP (RCP) far fields with polarizations ${\hat{e}}_{\mp }=(\hat{x}\mp i\hat{y})/\sqrt{2}$ on the top of the cavity, respectively. On the other hand, modes with $\chi =\pm 1$ have vanishing far fields above the cavity and contribute no CP radiation in the scheme of normal detection. It is necessary to ensure that modes with $\chi =\pm 1$ do not stand out in the presence of gain.

The LCP- and RCP-like modes are degenerate in 4-fold rotationally symmetric cavities. The degeneracy originates from the reciprocity and is analogous to the TR partner states in quantum mechanical systems with the *n*-fold rotation symmetry ($n\ge 3$). The two degenerate modes have the same resonant wavelengths and quality factors. They not only experience the same loss or gain but also receive identical amounts of spontaneous emissions. The chirality does not pick up the lasing action of either LCP- or RCP-like mode in a 4-fold rotationally symmetric cavity. However, it marks two types of modes off in the far field. The modal fields ${{\bf{E}}}_{{\rm{L}}}({\bf{r}})$ and ${{\bf{E}}}_{{\rm{R}}}({\bf{r}})$ of LCP- and RCP-like modes behave as ${a}_{{\rm{L}}}{\hat{e}}_{-}$ and ${a}_{{\rm{R}}}{\hat{e}}_{+}$ in the far-field zone above the gammadions, respectively, where ${a}_{{\rm{L}}}$ and ${a}_{{\rm{R}}}$ are the CP amplitudes. If the electric energies of the two modal fields in the GaN post are set equal for fair comparisons, the two magnitudes $|{a}_{{\rm{L}}}|$ and $|{a}_{{\rm{R}}}|$ can be distinct. Therefore, even if the two modes lase simultaneously with identical weights, the overall far field may still exhibit a non-vanishing dissymmetry factor ${g}_{{\rm{e}}}^{(4{\rm{f}})}$:

2 $${g}_{{\rm{e}}}^{({\rm{4f}})}=2\frac{|{a}_{{\rm{L}}}{|}^{2}-|{a}_{{\rm{R}}}{|}^{2}}{|{a}_{{\rm{L}}}{|}^{2}+|{a}_{{\rm{R}}}{|}^{2}}$$

where the superscript “4 f” means an ideal 4-fold rotationally symmetric cavity. A gammadion in experiment is seldom 4-fold rotationally symmetric. Any perturbations that break the 4-fold rotation symmetry lift the degeneracy of two CP-like modes. However, if the reciprocity is preserved, and the permittivity variation perturbs the two CP-like modes evenly, the two modal fields ${{\bf{E}}}_{{\rm{L}}}({\bf{r}})$ and ${{\bf{E}}}_{{\rm{R}}}({\bf{r}})$ are mixed into ${{\bf{E}}}_{1}({\bf{r}})$ and ${{\bf{E}}}_{2}({\bf{r}})$ of perturbed modes with equal weights, namely,

3 $${{\bf{E}}}_{(1,2)}({\bf{r}})\approx \frac{1}{\sqrt{2}}[{{\bf{E}}}_{{\rm{R}}}({\bf{r}})\pm {e}^{i\Theta }{{\bf{E}}}_{{\rm{L}}}({\bf{r}})]$$

where $\Theta$ is a phase factor (see section I, Supplementary Information). The far fields of ${{\bf{E}}}_{1}({\bf{r}})$ and ${{\bf{E}}}_{2}({\bf{r}})$ resemble $[{a}_{{\rm{R}}}{\hat{e}}_{+}\pm \exp (i\Theta ){a}_{{\rm{L}}}{\hat{e}}_{-}]/\sqrt{2}$ and both have a dissymmetry factor of ${g}_{{\rm{e}}}^{(4{\rm{f}})}$. These two modes are no longer degenerate. No matter which of them dominates in lasing, the far field exhibits a nonzero dissymmetry factor.

**Figure 2:**
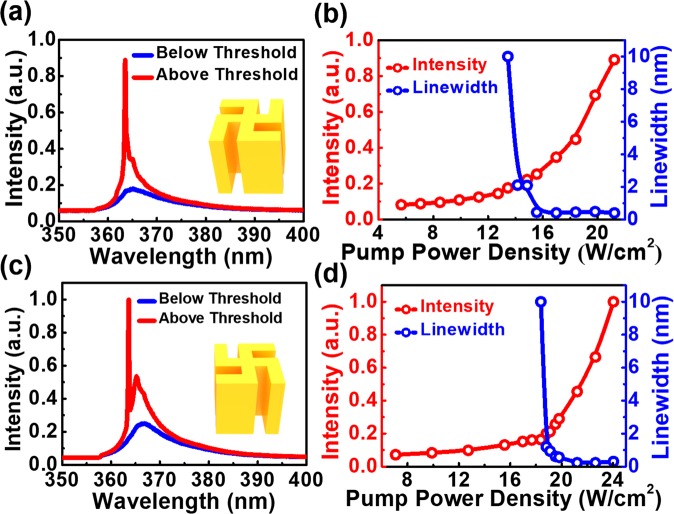
The lasing characteristics of the R- and L-gammadion metal cavities. (**a**) The PL spectra of the R-gammadion below (blue) and above (red) thresholds and (**b**) corresponding lasing intensity and linewidth as a function of the pump power density. The counterparts of the L-gammadion are presented in (**c**,**d**), respectively.

In experiment, the array of gammadion cavities was excited by a 355 nm pulse laser at room temperature (see Methods). The lasing signal of the R-gammadion at 364 nm is shown in Fig. 2a. Its light-in and light-out (L-L) curve in Fig. 2b indicates a threshold density of 14.9 W/cm^2^. The significant linewidth narrowing from several nm down to 0.35 nm when pump power density exceeded the lasing threshold also supported the lasing action from the metal-GaN gammadion cavity. Similar lasing behaviors were also observed from the L-gammadion cavity. From the PL spectra of L-gammadions in Fig. 2c, a lasing peak around 364 nm was also present above the threshold at room temperature. The L-L curve in Fig. 2d indicates a similar threshold of approximately 18.8 W/cm^2^ to that of the R-gammadion, and the linewidth narrowing also occurred around the threshold. The small threshold difference between the R- and the L-gammadion cavity lasers was attributed to the fabrication imperfection of the cavities. The lasing properties were also insensitive to periods of the arrays (see section II, Supplementary Information). This phenomenon indicates that the lasing actions in each gammadion were independent of each due to the isolation from metal coating.

**Figure 3:**
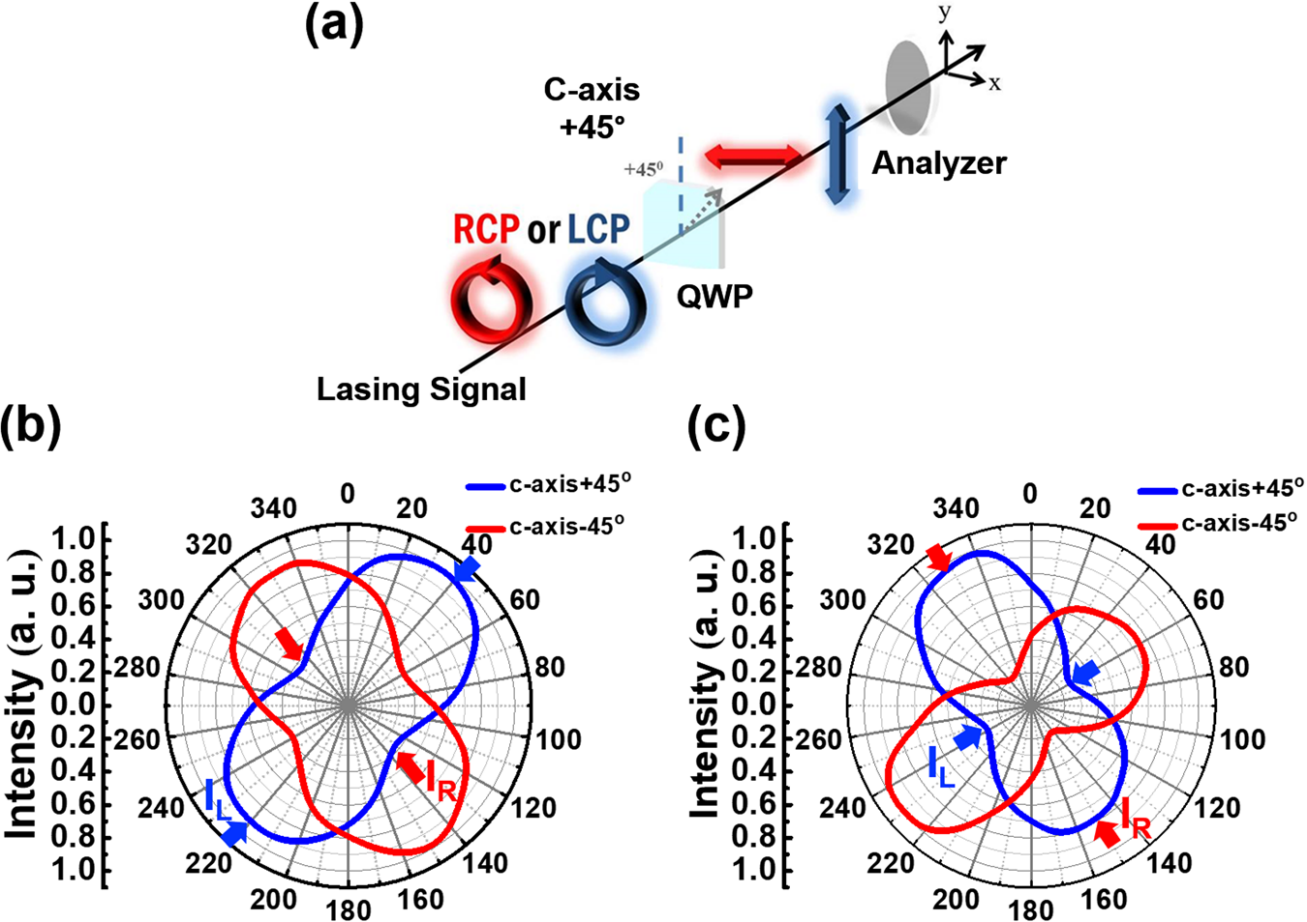
The quantification of polarization states for the outputs from R- and L-gammadion cavities. (**a**) The schematic diagram for CP analyzer. The angular distributions of polarized intensity behind the linear polarizer for the (**b**) R- and (**c**) L-gammadion cavities, respectively.

The circular dichroism is characterized to quantify the polarization states of output fields. A tunable quarter-wave ($\lambda /4$) plate and a linear polarizer were added into the setup depicted in Fig. 3a. The $\lambda /4$ plate converted the RCP/LCP signals into two orthogonal linearly polarized (LP) counterparts. By tuning the rotation angle of the following linear polarizer, we obtained the difference of the two CP components ${I}_{{\rm{R}}}$ and ${I}_{{\rm{L}}}$. Comparing these two CP components, we assess whether the laser output was RCP- or LCP-like through the experimental dissymmetry factors $\,{g}_{{\rm{e}}}$:

4 $${g}_{{\rm{e}}}=2\frac{{I}_{{\rm{L}}}-{I}_{{\rm{R}}}}{{I}_{{\rm{L}}}+{I}_{{\rm{R}}}}$$

A perfect CP state has maximal modulus $|{g}_{{\rm{e}}}|$ of 2, and a positive (negative) dissymmetry factor indicates that the output radiation is LCP-like (RCP-like). Figure 3(b,c) show angular distributions of polarized intensity from the R- and L-gammadions recorded behind the linear polarizer, respectively. The corresponding ${g}_{{\rm{e}}}$ are about 1 and −1.1, respectively. It appears that at the metal side, the handedness of lasing output was opposite to the orientation of gammadion arms, implying some conservation between the optical handedness and structural chirality. We expect that the higher dissymmetry factor could be obtained with the advanced near-field characterization and the further design of the nanocavity.

**Figure 4:**
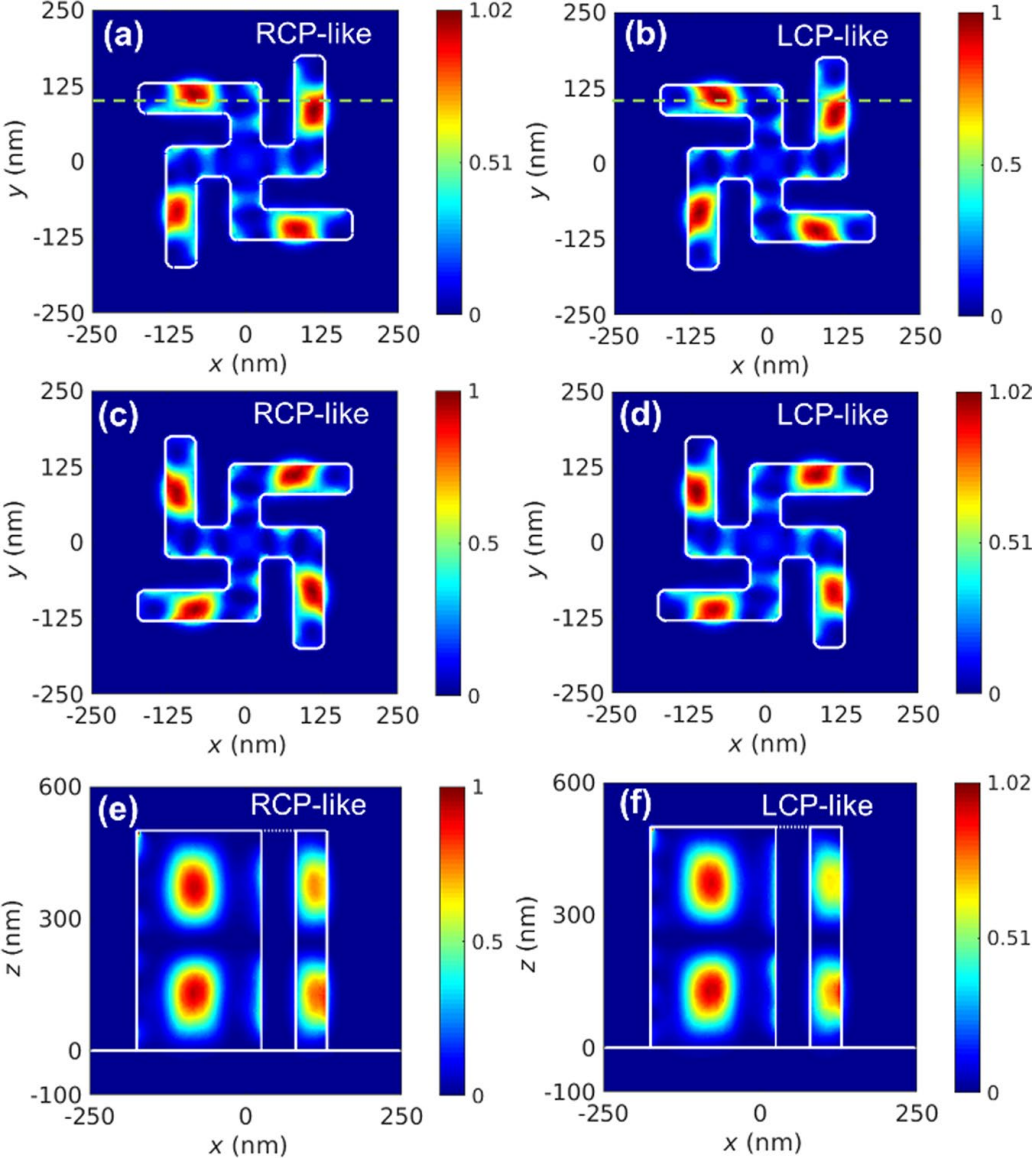
The intensity profiles of modal fields in an R- gammadion cavity. Horizontal intensity profiles $|{\bf{E}}({\bf{r}}){|}^{2}$ of (**a**) RCP- and (**b**) LCP-like lasing modes in an ideal R-gammadion metal cavity. Profiles in (**c**,**d**) are the counterparts of (**a**,**b**) in an ideal L-gammadion. Vertical intensity profiles $|{\bf{E}}({\bf{r}}){|}^{2}$ of the (**e**) RCP- and (**f**) LCP-like lasing modes in the ideal R-gammadion which pass through the green dashed lines in (**a**,**b**). Above numerical calculations are performed on COMSOL Multiphysics 5.3.

We use the finite-element method (commercial software package COMSOL Multiphysics) to find potential CP-like lasing modes (${\rm{\chi }}=\pm i$) in ideal gammadions around the gain window of GaN. Modes with real characters ${\rm{\chi }}=\pm 1$ are also examined, and their lasing conditions are not as favorable as those of CP-like modes (see section III, Supplementary Information). Figure 4a,b show intensity profiles $|{\bf{E}}({\bf{r}}){|}^{2}$ of the RCP- and LCP-like lasing modes on a horizontal cross section of the R-gammadion metal cavity, respectively. The counterparts of L-gammadions are presented in Fig. 4c,d. The resonance wavelengths of all these modes are the same and around 362.8 nm, which is close to 364 nm in the experiment. These modes behave as standing waves of the guided modes in gammadion waveguides, and the profiles in Fig. 4a,d are taken at an antinode. The vertical standing-wave patterns of the RCP- and LCP-like modes in the R-gammadion, which pass through the green dashed lines in Fig. 4a,b, are depicted in Fig. 4e,f, respectively. All the intensity profiles of CP-like modes exhibit the 4-fold rotation symmetry, and the patterns of RCP- and LCP-like modes look similar in either case of the gammadions. There are bright spots located in the four arms but dim fields around the center of gammadions. The local transverse fields inside cavities are far from circularly-polarized, but the far fields above the gammadions are. The perfect CP radiation of these CP-like modes atop of the cavity is due to far-field interferences of the local fields on neighboring arms of metal-coated gammadions, whose polarizations and amplitudes are rotated consecutively by $\pi /2$ in the real space and shifted $\,\pm \pi /2$ in phase, respectively. Since reciprocal perturbations that evenly affect the two CP-like modes tend to mix them with equal weights, we could estimate the dis-symmetry factor without details of perturbations in experiment. We numerically prolong one outer arm of the R-gammadion metal cavity by 3%, which have similar effects on the two CP-like modes whose spatial profiles look alike (see section IV, Supplementary Information). If the mixing with modes with ${\rm{\chi }}=\pm 1$ is minor, we obtain a dissymmetry factor of 0.92 for the perturbed mode, which is close to ${g}_{{\rm{e}}}=1$ in the experiment.

Experimentally, we further converted the LP output of pump laser into a CP beam to excite the R-gammadion cavity. The threshold power under the RCP pump was much lower than that under the LCP counterpart (see section V, Supplementary Information). This result is consistent with the reciprocity theorem and handedness of output field (${\hat{e}}_{-}$-like). The Lorentz reciprocity indicates that the RCP pump which may be regarded as being radiated by an ${\hat{e}}_{+}$-polarized source on the top of R-gammadion induces the more intense field inside the cavity than the LCP pump does. More electron-hole pairs are generated in this way, and therefore the threshold is lowered. The local photon density of states in the gammadion posts could also be modified by the plasmonic structure to increase the spontaneous emission rate. The enhanced transition rates stem from small modal volumes $\,V$ of the modes in the plasmonic cavity. We obtain a dimensionless modal volume ${V}_{{\rm{eff}}}=V{(\lambda /n)}^{-3}\approx 2.56$ for the LCP-like mode in an ideal R-gammadion metal cavity, where $\lambda$ is the wavelength of the mode; and $\,n$ is the refractive index of GaN (see section VI, Supplementary Information)^43^. This number indicates that the spontaneous-emission rate in a gammadion metal cavity is enhanced noticeably.

**Figure 5:**
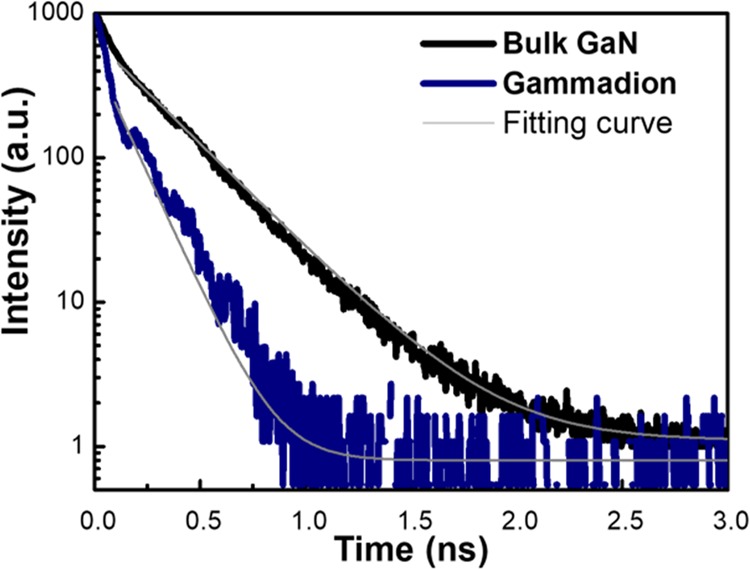
The TRPL traces of the bulk GaN and R-gammadions. The carrier lifetimes corresponding to the former and latter are 300 and 130 ps, respectively.

To estimate the Purcell factor *F* of the nanocavity, we measured the carrier lifetime with the time-resolved photoluminescence (TRPL). The traces of TRPL corresponding to the bulk GaN and R-gammadions below thresholds are shown in Fig. 5. We estimate their carrier lifetimes by fitting the time evolutions with one-component exponential decay. The extracted carrier lifetime ${\tau }_{{\rm{b}}}$ of bulk GaN and counterpart ${\tau }_{\Gamma }$ of the gammadions are 300 and 130 ps, respectively. The reciprocals of ${\tau }_{{\rm{b}}}$ and ${\tau }_{\Gamma }$ can be expressed as

5 $$\frac{1}{{\tau }_{{\rm{b}}}}=\frac{1}{{\tau }_{{\rm{r}}}}+\frac{1}{{\tau }_{{\rm{b}},{\rm{nr}}}},\,\frac{1}{{\tau }_{\varGamma }}=\frac{F}{{\tau }_{r}}+\frac{1}{{\tau }_{\varGamma ,{\rm{nr}}}}$$

where ${\tau }_{{\rm{r}}}$ and ${\tau }_{{\rm{nr}}}$ are the radiative and non-radiative recombination lifetimes of bulk GaN; *F* is the Purcell factor; and ${\tau }_{\Gamma ,{\rm{nr}}}$ is the non-radiative recombination lifetime in gammadions. For our samples, the non-radiative recombination rate was much smaller than the radiative one and could be dropped, and we approximate ${\tau }_{{\rm{b}}}^{-1}\approx {\tau }_{{\rm{r}}}^{-1}\,=$3.33 ns^−1^. For gammadion cavities, we similarly estimate the enhanced recombination rate as ${\tau }_{\Gamma }^{-1}\approx F/{\tau }_{{\rm{r}}}\,=$ 7.69 ns^−1^. In this way, the Purcell factor *F* is about 2.3, indicating that the faster response of gammadion metal-cavity lasers than that of typical GaN laser diodes could be potentially achieved.

### Summary

In conclusions, we have demonstrated chiral nanolasers based on metal-coated GaN gamma-dions at UV wavelengths, which can lase with a high degree of circular polarization at room temperature. Both the R- and L-gammadion lasers exhibited dissymmetry factors with $|{g}_{{\rm{e}}}|$ no less than unity. While the chirality did not pick up the LCP-like or RCP-like mode, it introduced handedness into far fields of lasing signals. These chiral nanolasers with ultra-small footprint could be adopted for CP photon applications in chip-scale integrated systems.

## Methods

### Device fabrication

We used 2-μm-thick undoped GaN as the gain medium of the chiral nanolaser, which was grown on a c-plane (0001) sapphire substrate with a metal-organic chemical vapor deposition system (EMCORE D-75). During the growth, trimethyl gallium (TMGa) and ammonia (NH_3_) were utilized as the gallium and nitride sources, respectively. An athermal process was used at 1080 °C for 10 minutes to clean the sample surface in a stream of ambient hydrogen before the epitaxial layers were grown. A 30-nm-thick GaN nucleation layer was then grown on the sapphire substrate at 530 °C, and the 2-μm-thick undoped GaN layer was then grown on it at 1040 °C. After the undoped sample was prepared, a 300-nm-thick Si_3_N_4_ layer was deposited on the GaN layer as an etching mask through the plasma-enhanced chemical vapor deposition. Next, we coated 250-nm negative photoresist (ma-N 2403) on the Si_3_N_4_ layer with spin coating. The gammadion pattern was first defined on the layer of negative photoresist through the E-beam lithography, after which the reactive ion etching (RIE) with a CHF_3_/O_2_ mixture was carried out to etch down to the Si_3_N_4_ layer. Next, we transferred the gammadion pattern from the Si_3_N_4_ layer to the undoped GaN layer to form the chirality structure (about 500-nm deep) through inductively coupled plasma RIE with a Cl_2_/Ar mixture. The Si_3_N_4_ mask layers were removed by wet etching after all the mentioned processes were completed. We then cleaned our sample by wet etching, washing away the particles created in previous processes of dry etching to enhance the performance of our device. Subsequently, a 50 nm aluminum layer was coated on the device by E-gun evaporation to form the planar chiral metal cavity based on GaN gammadions.

### Optical characterization

To measure lasing characteristics of the devices, the GaN gammadion metal cavities were optically pumped with a frequency- tripled 355-nm Nd:YVO_4_ pulse laser at room temperature. The pulse width and repetition rate of the laser were 0.5 ns and a 1 kHz, respectively. The spot size of the normally incident beam was approximately 30 μm. A 100× objective lens was used to collect the lasing signal from the chiral nanolasers through a multimode fiber which coupled the signal into a spectrometer with a detector of charge-coupled devices. The nanocavities were pumped from the top to prevent the absorption from the thick GaN layer beneath the device. For TRPL measurements, the center wavelength, pulse width, and repetition rate of the third-harmonic generation from the Ti:sapphire pulse laser were 266 nm, 200 fs, and 76 MHz, respectively. The response time of photo- multiplier tube was shorter than 50 ps.

## Supplementary information

supplementary information.
