# Supplementary material for: High Circular Polarized Nanolaser with Chiral Gammadion Metal Cavity
Source: Sci Rep. 2020 May 12;10:7880. doi: 10.1038/s41598-020-64836-1 (PMC7217972; doi:10.1038/s41598-020-64836-1)
Supplement: Supplementary file 1 — supplementary information. [file 41598_2020_64836_MOESM1_ESM.pdf]

Supplementary Information

High Circular Polarized Nanolaser with Chiral Gammadion Metal Cavity

Cheng-Li Yu[1,2], Yu-Hao Hsiao[1], Chiao-Yun Chang[2], Pi-Ju Cheng[2], Hsiang-Ting Lin[2], Ming-Sheng Lai[1,2], Hao-Chung Kuo[1], Shu-Wei Chang[1,2], and Min-Hsiung Shih[1,2,3]*

[1]Department of Photonics and Institute of Electro-Optical Engineering, National Chiao Tung University, Hsinchu 30010, Taiwan

[2]Research Center for Applied Sciences, Academia Sinica, Taipei 11529, Taiwan

[3]Department of Photonics, National Sun Yat-sen University, Kaohsiung 80424, Taiwan

*E-mail address: mhshih@gate.sinica.edu.tw

I. Weights of Two Circularly-Polarized-Like Modes in Perturbed Counterparts under Reciprocal Perturbations of Four-Fold Symmetric Cavities

The wave equation governing the unperturbed modal electric field $\mathbf{E}^{(0)}(\mathbf{r})$ which represents $\mathbf{E}_{\mathrm{R}}(\mathbf{r})$ and $\mathbf{E}_{\mathrm{L}}(\mathbf{r})$ of the RCP- and LCP-like modes, respectively, in a 4-fold rotationally symmetric cavity can be expressed as

$$\nabla \times \nabla \times \mathbf{E}^{(0)}(\mathbf{r}) - \left(\frac{\omega_{\mathrm{r}}}{c}\right)^2 \bar{\bar{\epsilon}}_{\mathrm{r}}^{(0)}(\mathbf{r}, \omega_{\mathrm{r}})\mathbf{E}^{(0)}(\mathbf{r}) = \mathbf{0}, \tag{S1.1a}$$

$$\mathbf{R}_{\pi/2}\bar{\bar{\epsilon}}_{\mathrm{r}}\left(\mathbf{R}_{\pi/2}^{-1}\mathbf{r}, \omega_{\mathrm{r}}\right)\mathbf{R}_{\pi/2}^{-1} = \bar{\bar{\epsilon}}_{\mathrm{r}}(\mathbf{r}, \omega_{\mathrm{r}}), \tag{S1.1b}$$

$$\mathbf{R}_{\pi/2} = \begin{pmatrix} 0 & -1 & 0 \\ 1 & 0 & 0 \\ 0 & 0 & 1 \end{pmatrix}, \tag{S1.1c}$$

where c is the speed of light in vacuum; $\bar{\bar{\epsilon}}_{\mathrm{r}}^{(0)}(\mathbf{r}, \omega_{\mathrm{r}})$ is the relative permittivity tensor corresponding to the cavity with gain and exhibits the 4-fold rotation symmetry in Eq. (S1.1b); $\mathbf{R}_{\pi/2}$ is the matrix associated the rotation around the z axis by $\pi/2$; and ω_{r} is the (real) resonant frequency of the two CP-like modes at the threshold. For fair comparisons, we demand that the integrals of square field magnitudes associated with

the two CP-like modes in the 4-fold rotationally symmetric active region $\Omega_{\mathrm{a}}^{(0)}$ (assumed to be composed of isotropic gain medium) to be identical, namely,

$$\int_{\Omega_{\mathrm{a}}^{(0)}} \mathrm{d}\mathbf{r}\,|\mathbf{E}_{\mathrm{R}}(\mathbf{r})|^2 = \int_{\Omega_{\mathrm{a}}^{(0)}} \mathrm{d}\mathbf{r}\,|\mathbf{E}_{\mathrm{L}}(\mathbf{r})|^2.$$

(S1.2)

In the presence of a perturbation $\Delta\bar{\bar{\epsilon}}_{\mathrm{r}}(\mathbf{r},\omega)$ of relative permittivity tensor which is reciprocal $[\Delta\bar{\bar{\epsilon}}_{\mathrm{r}}^{\mathrm{T}}(\mathbf{r},\omega) = \Delta\bar{\bar{\epsilon}}_{\mathrm{r}}(\mathbf{r},\omega)]$ but breaks the 4-fold rotation symmetry at a new resonance frequency ω, the overall permittivity $\bar{\bar{\epsilon}}_{\mathrm{r}}(\mathbf{r},\omega)$ which describes fields $\mathbf{E}(\mathbf{r})$ of perturbed modes [including the nondegenerate ones $\mathbf{E}_1(\mathbf{r})$ and $\mathbf{E}_2(\mathbf{r})$ originating from $\mathbf{E}_{\mathrm{R}}(\mathbf{r})$and $\mathbf{E}_{\mathrm{L}}(\mathbf{r})$] at new thresholds is

$$\bar{\bar{\epsilon}}_{\mathrm{r}}(\mathbf{r},\omega) = \bar{\bar{\epsilon}}_{\mathrm{r}}^{(0)}(\mathbf{r},\omega) + \Delta\bar{\bar{\epsilon}}_{\mathrm{r}}(\mathbf{r},\omega) + \Delta\epsilon_{\mathrm{r,a}}U(\mathbf{r})\bar{\bar{I}},$$

(S1.3)

where $\Delta\epsilon_{\mathrm{r,a}}$ is an imaginary number representing the required adjustment of relative permittivity in the active region which makes the mode self-oscillate (real ω) with a constant magnitude in the perturbed environment; $U(\mathbf{r})$ is an indicator function which is unity in the active region Ω_{a} but zero elsewhere; and $\bar{\bar{I}}$ is the 3-by-3 identity tenor. For simplicity, we assume that $\Delta\bar{\bar{\epsilon}}_{\mathrm{r}}(\mathbf{r},\omega)$ is only present in a finite region around the cavity. Also note that the indicator function $U(\mathbf{r})$ of the active region Ω_{a} need not be

4-fold symmetric in the presence of perturbations, in contrast to the counterpart $U^{(0)}(\mathbf{r})$ of the original active region $\Omega_{\mathrm{a}}^{(0)}$ which has the 4-fold rotation symmetry. In analogy to Eq. (S1.1a), the wave equation for $\mathbf{E}(\mathbf{r})$ is written as

$$\nabla \times \nabla \times \mathbf{E}(\mathbf{r}) - \left(\frac{\omega}{c}\right)^2 \bar{\bar{\epsilon}}_{\mathrm{r}}(\mathbf{r}, \omega)\mathbf{E}(\mathbf{r}) = \mathbf{0}. \tag{S1.4}$$

We then rewrite Eq. (S1.4) in a perturbative manner. The frequency difference $\Delta\omega \equiv \omega - \omega_{\mathrm{r}}$ and permittivity variation $\Delta\epsilon_{\mathrm{r,a}}$ should vary linearly with $\Delta\bar{\bar{\epsilon}}_{\mathrm{r}}(\mathbf{r}, \omega_{\mathrm{r}})$. Therefore, to the order $\mathrm{O}[\Delta\bar{\bar{\epsilon}}_{\mathrm{r}}(\mathbf{r}, \omega_{\mathrm{r}})]$, we recast Eq. (S1.4) in the following form:

$$\nabla \times \nabla \times \mathbf{E}(\mathbf{r}) - \left(\frac{\omega_{\mathrm{r}}}{c}\right)^2 \bar{\bar{\epsilon}}_{\mathrm{r}}^{(0)}(\mathbf{r}, \omega_{\mathrm{r}})\mathbf{E}(\mathbf{r}) \approx \left[\Delta\bar{\bar{A}}(\mathbf{r}) + \frac{\Delta\omega}{\omega_{\mathrm{r}}}\bar{\bar{A}}^{(\mathrm{d})}(\mathbf{r}) + \Delta\epsilon_{\mathrm{r,a}}\bar{\bar{A}}^{(\mathrm{a})}(\mathbf{r})\right] \mathbf{E}(\mathbf{r}), \tag{S1.5a}$$

$$\Delta\bar{\bar{A}}(\mathbf{r}) = \left(\frac{\omega_{\mathrm{r}}}{c}\right)^2 \Delta\bar{\bar{\epsilon}}_{\mathrm{r}}(\mathbf{r}, \omega_{\mathrm{r}}), \quad \bar{\bar{A}}^{(\mathrm{d})}(\mathbf{r}) = \omega_{\mathrm{r}} \frac{\partial}{\partial\omega'}\left[\left(\frac{\omega'}{c}\right)^2 \bar{\bar{\epsilon}}_{\mathrm{r}}^{(0)}(\mathbf{r}, \omega')\right]_{\omega'=\omega_{\mathrm{r}}},$$

$$\bar{\bar{A}}^{(\mathrm{a})}(\mathbf{r}) = \left(\frac{\omega_{\mathrm{r}}}{c}\right)^2 U(\mathbf{r})\bar{\bar{I}}, \tag{S1.5b}$$

where $\Delta\bar{\bar{A}}(\mathbf{r})$, $\bar{\bar{A}}^{(\mathrm{d})}(\mathbf{r})$, and $\bar{\bar{A}}^{(\mathrm{a})}(\mathbf{r})$ are tensors featuring variations due to $\Delta\bar{\bar{\epsilon}}_{\mathrm{r}}(\mathbf{r}, \omega_{\mathrm{r}})$ (permittivity perturbation), $\Delta\omega/\omega_{\mathrm{r}}$ (fractional frequency shift due to perturbation or dispersion), and $\Delta\epsilon_{\mathrm{r,a}}$ (extrinsic permittivity variation in the active region so as to

make $\Delta\omega$ real), respectively. In Eq. (S1.5a), it is $\Delta\omega/\omega_\mathrm{r}$ and $\Delta\epsilon_\mathrm{r,a}$ that need to be determined in successive calculations.

We may now expand the field $\mathbf{E}(\mathbf{r})$ as follows:

$$\mathbf{E}(\mathbf{r}) = C_\mathrm{R}\mathbf{E}_\mathrm{R}(\mathbf{r}) + C_\mathrm{L}\mathbf{E}_\mathrm{L}(\mathbf{r}) + \Delta\mathbf{E}(\mathbf{r}), \tag{S1.6}$$

where C_R and C_L are the zeroth-order expansion coefficients of $\mathbf{E}_\mathrm{R}(\mathbf{r})$ and $\mathbf{E}_\mathrm{L}(\mathbf{r})$, respectively; and $\Delta\mathbf{E}(\mathbf{r})$ is a perturbed field of the second order in $\Delta\bar{\bar{\epsilon}}_\mathrm{r}(\mathbf{r},\omega_\mathrm{r})$, that is, $\Delta\mathbf{E}(\mathbf{r}) = \mathrm{O}[\Delta\bar{\bar{\epsilon}}_\mathrm{r}^2(\mathbf{r},\omega_\mathrm{r})]$. Substituting Eq. (S1.6) into Eq. (S1.5a), we obtain the relation:

$$\left[\Delta\bar{\bar{A}}(\mathbf{r}) + \frac{\Delta\omega}{\omega_\mathrm{r}}\bar{\bar{A}}^{(\mathrm{d})}(\mathbf{r}) + \Delta\epsilon_\mathrm{r,a}\bar{\bar{A}}^{(\mathrm{a})}(\mathbf{r})\right][C_\mathrm{R}\mathbf{E}_\mathrm{R}(\mathbf{r}) + C_\mathrm{L}\mathbf{E}_\mathrm{L}(\mathbf{r})] = \mathrm{O}[\Delta\bar{\bar{\epsilon}}_\mathrm{r}^2(\mathbf{r},\omega_\mathrm{r})]. \tag{S1.7}$$

We then project Eq. (S1.7) into the subspace spanned by $\mathbf{E}_\mathrm{R}(\mathbf{r})$ and $\mathbf{E}_\mathrm{L}(\mathbf{r})$. For this purpose, an integration region Ω with the 4-fold rotation symmetry needs to be picked up to define the inner product. Here, we simply choose Ω as the minimal circular cylinder that completely covers the perturbation $\Delta\bar{\bar{\epsilon}}_\mathrm{r}(\mathbf{r},\omega)$. In this way, a 2-by-2 matrix equation for column vector $\mathbf{C} = (C_\mathrm{R}, C_\mathrm{L})^\mathrm{T}$ (superscript "T" means transpose) can be constructed:

$$\left[\Delta\mathbf{A} + \Delta\epsilon_{\mathrm{r,a}}\mathbf{A}^{(\mathrm{a})}\right]\mathbf{C} = -\frac{\Delta\omega}{\omega_{\mathrm{r}}}\mathbf{A}^{(\mathrm{d})}\mathbf{C},$$

(S1.8a)

where $\Delta\mathbf{A}$, $\mathbf{A}^{(\mathrm{a})}$, and $\mathbf{A}^{(\mathrm{d})}$ are 2-by-2 matrix representations of $\Delta\bar{\bar{A}}(\mathbf{r})$, $\bar{\bar{A}}^{(\mathrm{d})}(\mathbf{r})$, and $\bar{\bar{A}}^{(\mathrm{a})}(\mathbf{r})$, respectively. Their matrix elements are defined as

$$\Delta\mathrm{A}_{\alpha\beta} = \int_{\Omega} \mathrm{d}\mathbf{r}\, \mathbf{E}_{\alpha}^{\mathrm{T}}(\mathbf{r})\Delta\bar{\bar{A}}(\mathbf{r})\mathbf{E}_{\beta}(\mathbf{r}) = \left(\frac{\omega_{\mathrm{r}}}{c}\right)^{2} \int_{\Omega} \mathrm{d}\mathbf{r}\, \mathbf{E}_{\alpha}^{\mathrm{T}}(\mathbf{r})\Delta\bar{\bar{\epsilon}}_{\mathrm{r}}(\mathbf{r},\omega_{\mathrm{r}})\mathbf{E}_{\beta}(\mathbf{r}),$$

$$\mathrm{A}_{\alpha\beta}^{(\mathrm{a})} = \int_{\Omega} \mathrm{d}\mathbf{r}\, \mathbf{E}_{\alpha}^{\mathrm{T}}(\mathbf{r})\bar{\bar{A}}^{(\mathrm{a})}(\mathbf{r})\mathbf{E}_{\beta}(\mathbf{r}) = \left(\frac{\omega_{\mathrm{r}}}{c}\right)^{2} \int_{\Omega} \mathrm{d}\mathbf{r}\, \mathbf{E}_{\alpha}^{\mathrm{T}}(\mathbf{r})U(\mathbf{r})\bar{\bar{I}}\mathbf{E}_{\beta}(\mathbf{r}),$$

$$\mathrm{A}_{\alpha\beta}^{(\mathrm{d})} = \int_{\Omega} \mathrm{d}\mathbf{r}\, \mathbf{E}_{\alpha}^{\mathrm{T}}(\mathbf{r})\bar{\bar{A}}^{(\mathrm{d})}(\mathbf{r})\mathbf{E}_{\beta}(\mathbf{r}) =$$

$$\omega_{\mathrm{r}} \int_{\Omega} \mathrm{d}\mathbf{r}\, \mathbf{E}_{\alpha}^{\mathrm{T}}(\mathbf{r}) \frac{\partial}{\partial\omega'}\left[\left(\frac{\omega'}{c}\right)^{2} \bar{\bar{\epsilon}}_{\mathrm{r}}^{(0)}(\mathbf{r},\omega')\right]_{\omega'=\omega_{\mathrm{r}}} \mathbf{E}_{\beta}(\mathbf{r}),$$

(S1.8b)

where indices α and β refer to R or L. Note that in Eq. (S1.8b), we do not use complex-conjugated vectors of $\mathbf{E}_{\mathrm{R}}(\mathbf{r})$ and $\mathbf{E}_{\mathrm{L}}(\mathbf{r})$ to define the inner product since the tensors $\Delta\bar{\bar{A}}(\mathbf{r})$ and $\bar{\bar{A}}^{(\mathrm{d})}(\mathbf{r})$ are in general non-Hermitian in the presence of loss and gain.

For a generic tensor $\bar{\bar{T}}(\mathbf{r})$ with the 4-fold rotation symmetry, it can be shown that in the corresponding 2-by-2 matrix representation $\mathbf{T}$, only the off-diagonal elements T_{RL} and T_{LR} could exist. The diagonal ones T_{RR} and T_{LL} have to vanish due to the 4-fold rotation symmetry. Furthermore, if $\bar{\bar{T}}(\mathbf{r})$ is symmetric [$\bar{\bar{T}}^{\mathrm{T}}(\mathbf{r}) = \bar{\bar{T}}(\mathbf{r})$],

which is valid for reciprocal media, the matrix $\mathbf{T}$ is also symmetric, namely, T_{RL} and T_{LR} are identical. In views of these properties, the matrices $\Delta\mathbf{A}$, $\mathbf{A}^{(\mathrm{a})}$, and $\mathbf{A}^{(\mathrm{d})}$ can be simplified as follows:

$$\Delta\mathbf{A} = \begin{pmatrix} \Delta\mathrm{A}_{\mathrm{RR}} & \Delta\mathrm{A}_{\mathrm{RL}} \\ \Delta\mathrm{A}_{\mathrm{RL}} & \Delta\mathrm{A}_{\mathrm{LL}} \end{pmatrix}, \quad \mathbf{A}^{(\mathrm{a})} = \begin{pmatrix} \mathrm{A}_{\mathrm{RR}}^{(\mathrm{a})} & \mathrm{A}_{\mathrm{RL}}^{(\mathrm{a})} \\ \mathrm{A}_{\mathrm{RL}}^{(\mathrm{a})} & \mathrm{A}_{\mathrm{LL}}^{(\mathrm{a})} \end{pmatrix},$$

$$\mathbf{A}^{(\mathrm{d})} = \begin{pmatrix} 0 & \mathrm{A}_{\mathrm{RL}}^{(\mathrm{d})} \\ \mathrm{A}_{\mathrm{RL}}^{(\mathrm{d})} & 0 \end{pmatrix} = \mathrm{A}_{\mathrm{RL}}^{(\mathrm{d})}\boldsymbol{\sigma}_1, \quad \boldsymbol{\sigma}_1 = \begin{pmatrix} 0 & 1 \\ 1 & 0 \end{pmatrix},$$

(S1.9)

where $\boldsymbol{\sigma}_1$ is the first Pauli matrix. With an expression of $\mathbf{A}^{(\mathrm{d})}$ in Eq. (S1.9) which is proportional to $\boldsymbol{\sigma}_1$, we can convert Eq. (S1.8a) into the form of eigenvalue problems by multiplying both sides with $\boldsymbol{\sigma}_1$:

$$\frac{1}{\mathrm{A}_{\mathrm{RL}}^{(\mathrm{d})}} \begin{pmatrix} \Delta\mathrm{A}_{\mathrm{RL}} + \Delta\epsilon_{\mathrm{r,a}}\mathrm{A}_{\mathrm{RL}}^{(\mathrm{a})} & \Delta\mathrm{A}_{\mathrm{LL}} + \Delta\epsilon_{\mathrm{r,a}}\mathrm{A}_{\mathrm{LL}}^{(\mathrm{a})} \\ \Delta\mathrm{A}_{\mathrm{RR}} + \Delta\epsilon_{\mathrm{r,a}}\mathrm{A}_{\mathrm{RR}}^{(\mathrm{a})} & \Delta\mathrm{A}_{\mathrm{RL}} + \Delta\epsilon_{\mathrm{r,a}}\mathrm{A}_{\mathrm{RL}}^{(\mathrm{a})} \end{pmatrix} \begin{pmatrix} C_{\mathrm{R}} \\ C_{\mathrm{L}} \end{pmatrix} = -\frac{\Delta\omega}{\omega_{\mathrm{r}}} \begin{pmatrix} C_{\mathrm{R}} \\ C_{\mathrm{L}} \end{pmatrix}.$$

(S1.10)

In principle, we need to adjust $\Delta\epsilon_{\mathrm{r,a}}$ to make $\Delta\omega$ real. However, for a quick estimation, we may first drop $\Delta\epsilon_{\mathrm{r,a}}$ and treat $\Delta\omega$ as a complex quantity, whose imaginary part now characterizes how fast the mode decays or grows. Furthermore, if the permittivity variation $\Delta\bar{\bar{\epsilon}}_{\mathrm{r}}(\mathbf{r}, \omega_{\mathrm{r}})$ perturbs the RCP- and LCP-like modes with

similar magnitudes, namely,

$$\left|\int_{\Omega} \mathrm{d}\mathbf{r}\, \mathbf{E}_{\mathrm{R}}^{\mathrm{T}}(\mathbf{r}) \Delta\bar{\bar{\epsilon}}_{\mathrm{r}}(\mathbf{r},\omega_{\mathrm{r}}) \mathbf{E}_{\mathrm{R}}(\mathbf{r})\right| \approx \left|\int_{\Omega} \mathrm{d}\mathbf{r}\, \mathbf{E}_{\mathrm{L}}^{\mathrm{T}}(\mathbf{r}) \Delta\bar{\bar{\epsilon}}_{\mathrm{r}}(\mathbf{r},\omega_{\mathrm{r}}) \mathbf{E}_{\mathrm{L}}(\mathbf{r})\right|, \tag{S1.11}$$

which is usually the case for uncontrolled (random) perturbations, the two complex matrix elements $\Delta\mathrm{A}_{\mathrm{RR}}$ and $\Delta\mathrm{A}_{\mathrm{LL}}$ would have close moduli despite difference phases. In this condition, we may rewrite $\Delta\mathrm{A}_{\mathrm{RR}}$, $\Delta\mathrm{A}_{\mathrm{LL}}$, $\Delta\mathrm{A}_{\mathrm{RL}}$, and $\mathrm{A}_{\mathrm{RL}}^{(\mathrm{d})}$ in polar forms as

$$\Delta\mathrm{A}_{\mathrm{RR}} = |\Delta\mathrm{A}_{\mathrm{RR}}| e^{i\phi_{\mathrm{RR}}}, \quad \Delta\mathrm{A}_{\mathrm{LL}} = |\Delta\mathrm{A}_{\mathrm{RR}}| e^{i\phi_{\mathrm{LL}}},$$
$$\Delta\mathrm{A}_{\mathrm{RL}} = |\Delta\mathrm{A}_{\mathrm{RL}}| e^{i\phi_{\mathrm{RL}}}, \quad \mathrm{A}_{\mathrm{RL}}^{(\mathrm{d})} = \left|\mathrm{A}_{\mathrm{RL}}^{(\mathrm{d})}\right| e^{i\phi_{\mathrm{RL}}^{(\mathrm{d})}}, \tag{S1.12}$$

where ϕ_{RR}, ϕ_{LL}, ϕ_{RL}, and $\phi_{\mathrm{RL}}^{(\mathrm{d})}$ are the corresponding phases. In this way, the eigenvalue problem in Eq. (S1.10) is simplified as

$$\frac{e^{-i\phi_{\mathrm{RL}}^{(\mathrm{d})}}}{|\mathrm{A}_{\mathrm{RL}}^{(\mathrm{d})}|} \begin{pmatrix} |\Delta\mathrm{A}_{\mathrm{RL}}| e^{i\phi_{\mathrm{RL}}} & |\Delta\mathrm{A}_{\mathrm{RR}}| e^{i\phi_{\mathrm{LL}}} \\ |\Delta\mathrm{A}_{\mathrm{RR}}| e^{i\phi_{\mathrm{RR}}} & |\Delta\mathrm{A}_{\mathrm{RL}}| e^{i\phi_{\mathrm{RL}}} \end{pmatrix} \begin{pmatrix} C_{\mathrm{R}} \\ C_{\mathrm{L}} \end{pmatrix} = -\frac{\Delta\omega}{\omega_{\mathrm{r}}} \begin{pmatrix} C_{\mathrm{R}} \\ C_{\mathrm{L}} \end{pmatrix}, \tag{S1.13a}$$

and the two complex fractional frequency shifts $\Delta\omega_1/\omega_{\mathrm{r}}$ and $\Delta\omega_2/\omega_{\mathrm{r}}$ as well as their eigenvectors $\mathbf{C}_1$ and $\mathbf{C}_2$ are

$$\frac{\Delta\omega_1}{\omega_r} = -\frac{|\Delta A_{RL}|}{|A_{RL}^{(d)}|} e^{i\left(\phi_{RL}-\phi_{RL}^{(d)}\right)} + \frac{|\Delta A_{RR}|}{|A_{RL}^{(d)}|} e^{i\left[\frac{1}{2}(\phi_{RR}+\phi_{LL})-\phi_{RL}^{(d)}\right]},$$

$$\mathbf{C}_1 = \frac{1}{\sqrt{2}}\begin{pmatrix} 1 \\ e^{\frac{i}{2}(\phi_{LL}-\phi_{RR})} \end{pmatrix},$$

(S1.13b)

$$\frac{\Delta\omega_2}{\omega_r} = -\frac{|\Delta A_{RL}|}{|A_{RL}^{(d)}|} e^{i\left(\phi_{RL}-\phi_{RL}^{(d)}\right)} - \frac{|\Delta A_{RR}|}{|A_{RL}^{(d)}|} e^{i\left[\frac{1}{2}(\phi_{RR}+\phi_{LL})-\phi_{RL}^{(d)}\right]},$$

$$\mathbf{C}_2 = \frac{1}{\sqrt{2}}\begin{pmatrix} 1 \\ -e^{\frac{i}{2}(\phi_{LL}-\phi_{RR})} \end{pmatrix}.$$

(S1.13c)

We may define a perturbation-dependent phase shift Θ as

$$\Theta = \frac{\phi_{LL} - \phi_{RR}}{2}.$$

(S1.14)

Substituting Eqs. (S1.13b), (S1.13c), and (S1.14) into Eq. (S1.6), we then obtain the perturbed modal profiles $\mathbf{E}_1(\mathbf{r})$ and $\mathbf{E}_2(\mathbf{r})$ in Eq. (3) of the main text:

$$\mathbf{E}_{(1,2)}(\mathbf{r}) \approx \frac{1}{\sqrt{2}}\left[\mathbf{E}_R(\mathbf{r}) \pm e^{i\Theta}\mathbf{E}_L(\mathbf{r})\right].$$

(S1.15)

II. **Lasing from The Cavities with Different Periods**

To ensure that the lasing action indeed originates from a single gammadion metal-cavity rather than collectively from the gammadion metasurface, we characterized lasing properties of the structures with different periods. Three planar arrays of chiral nanolasers with periods of 600, 700, and 800 nm were fabricated and optically pumped under the same measurement condition. The corresponding SEM images of device with periods of 600, 700 and 800 nm and their PL spectra at a fixed pump power density were shown in Fig. S2(a), (b), and (c). The lasing peaks of these GaN gammadion metal cavities with different periods all occurred approximately at 364 nm at room temperature. Consequently, the experimental results indicate that lasing wavelength is independent of the period. In other words, the lasing action was mainly the result of a single gammadion metallic nanocavity.

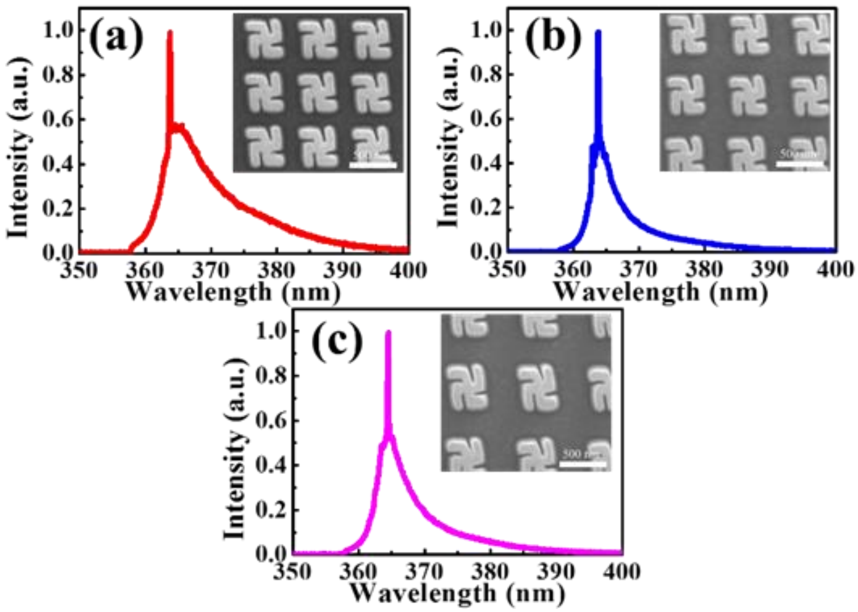

Figure S1. Lasing spectra of gammadion metal-cavities with different periods of (a) 600, (b) 700, and (c) 800 nm. The insets show the SEM images of corresponding GaN gammadion metal cavities.

III. Mode Analysis in Gammadion Metal Cavity

In addition to the degenerate CP-like modes, other cavity modes might be also present in the wavelength range around 364 nm. We need to justify that the target CP-like modes or their linear combinations were responsible for the lasing signal and circular dichroism in experiment. For this purpose, we calculate complex resonant frequencies ω_r of the modes at different negative imaginary part κ_a of the refractive index of GaN (gammadion post). The real parts $\mathrm{Re}[\omega_r]$ determine resonance wavelengths of the cavity modes. As κ_a turns more negative, the imaginary parts $\mathrm{Im}[\omega_r]$ become smaller, corresponding to the longer photon lifetimes. The magnitudes $|\kappa_a|$ at which the imaginary parts $\mathrm{Im}[\omega_r]$ vanish are proportional to the threshold modal gains. The larger $|\kappa_a|$ (more negative κ_a) indicate the higher thresholds.

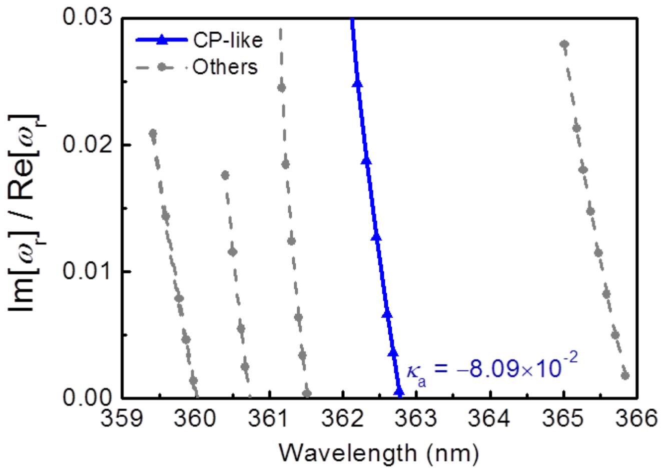

Figure S2. The ratios $\mathrm{Im}[\omega_r]/\mathrm{Re}[\omega_r]$ versus the resonance wavelength for various modes under different imaginary parts κ_a of the refractive index of GaN in the

gammadion post. The corresponding κ_a at thresholds, from the short- to long-wavelength side, are -8.21×10^{-2}, -7.91×10^{-2}, -8.06×10^{-2}, -8.09×10^{-2}, and -0.103, respectively. The ratios of potential CP-like lasing mode are marked with blue triangles.

In Figure S2, we show the ratios $\mathrm{Im}[\omega_r]/\mathrm{Re}[\omega_r]$ versus the resonance wavelength of 5 modes around the experimental lasing wavelength of 364 nm under different κ_a. Their imaginary parts of the refractive index of GaN at thresholds ($\mathrm{Im}[\omega_r] = 0$), from the short- to long-wavelength sides, are $\kappa_a = -8.21\times10^{-2}$, -7.91×10^{-2}, -8.06×10^{-2}, -8.09×10^{-2}, and -0.103, respectively. Experimentally, the material gain of GaN dropped rapidly at the short-wavelength side of 364 nm, leading to a narrow gain window. The modes presented in Figure 4 of the main context are the only CP-like ones in the window, and they reach the threshold at $\kappa_a = -8.09\times10^{-2}$ around a wavelength of 362.8 nm. All other modes have real characters $\chi = \pm1$ and are not circularly-polarized in the far-field zone above the gammadion. The ones with resonance wave-lengths shorter than 362.8 nm could have the lower thresholds than those of CP-like modes, but they fall out of the gain window and only receive small gain. On the other hand, the mode at the long-wavelength side (365.9 nm) could possibly lie in the gain window, but its threshold ($\kappa_a = -0.103$) is higher than that of CP-like modes, which is also unfavorable for lasing. Therefore, it is supported that the lasing signal in the experiment originated from the CP-like modes in gammadion metal cavities.

IV. Estimation of Dissymmetry Factor

We slightly lengthen an outer arm of the R-gammadion cavity by 3% to investigate the mixing of two CP-like modes. The horizontal field distributions of two perturbed modes (at the antinode) that are closely related to the two CP-like modes in an ideal gammadion metal cavity are depicted in Figure S3. The elongation of the arm may simply mix the two CP-like modes into a perturbed one, as indicated by the profile of y-like mode in Figure S3(a). On the other hand, the effect of higher-order perturbations could also come into play so that modes with real characters ($\chi = \pm 1$) are mixed into perturbed ones. Such a phenomenon takes place in the case of x-like mode whose horizontal field profile exhibits a hot spot in the outer arm at the bottom left, as shown in Figure S3(b).

We have calculated the dissymmetry factors of the far fields of these two modes based on the Fourier analysis of near fields just above the Gammadions. The y- and x-like modes exhibit dissymmetry factors of 0.92 and 0.8, respectively. Since the y-like mode is less affected by the coupling to modes with $\chi = \pm 1$, it should reflect the features of CP-like.

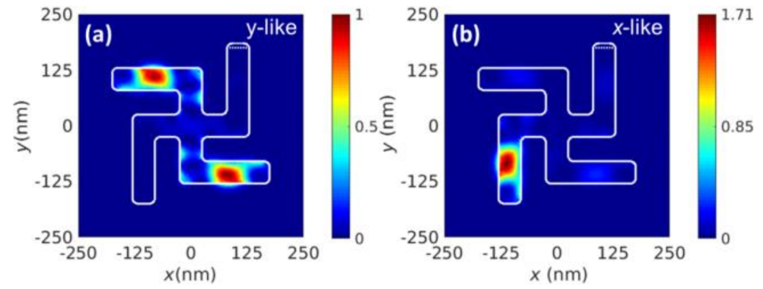

Figure S3. The horizontal field profiles of the (a) y- and (b) x-like modes at the antinode corresponding to a perturbed R-gammadion metal cavity. The outer arm indicated by white dashed lines is prolonged by 3%. The x-like one is significantly mixed with other modes with $\chi = \pm 1$. modes more faithfully than the x-like one would. Indeed, the dissymmetry factor of 0.92 associated with the former is close to the experimental counterpart $g_{\mathrm{e}} = 1$ observed from the lasing signal of R-gammadion cavities.

V. Cavity Pumping with Different Optical Polarizations

To further verify the polarization states of planar chiral nanolasers, right-handed gammadion metal cavities were optically pumped by the pulse laser with different polarization states including the linear, left-hand, and right-hand circular polarizations, as schematically illustrated in Fig. S4 (a). Figures S4(b) and (c) show the PL spectra and light-in and light-out (L-L) curve of the left-handed gammadion metal-cavity under different polarized pumping sources. From the L-L curve, the gammadion metal-cavity had the lower threshold power density of about 15.56 W/cm^2 in the presence of left-hand circularly polarized pumping. In contrast, we observed the higher threshold power density of about 18.39 W/cm^2 under right-hand circularly polarized pumping. This phenomenon indicated that the threshold of gammadion metal-cavities depends on the type of structure under different polarization states of the pumping source. The left-hand circularly polarized lasing output is favored by the right-handed gammadion metal-cavity and further boosted by the left-hand circularly-polarized pump, and similar trend also applies to the metal cavities and pump source with opposite handedness.

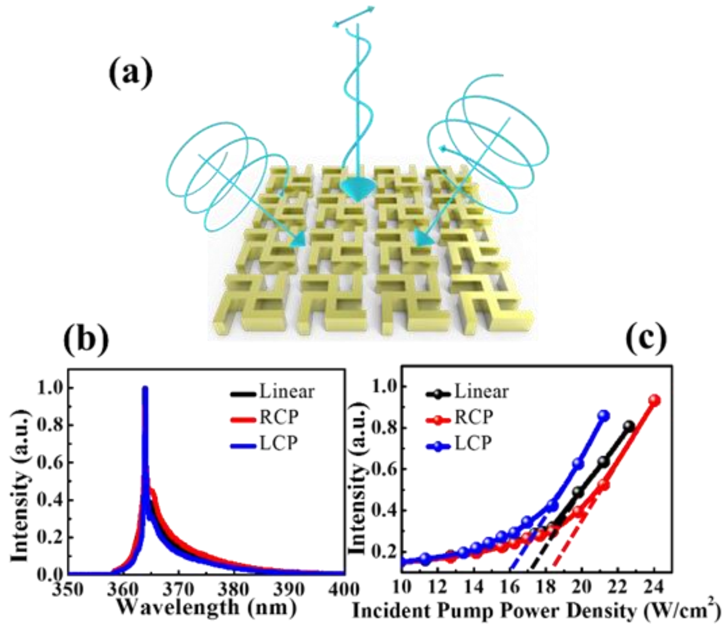

Figure S4. (a) Schematic diagram of different polarized pumping sources incident onto the right-handed gammadion metal cavity. (b) PL spectra and (c) L-L curves in different polarization conditions of pumping.

VI. Estimation of Modal Volume

The FEM was applied to investigate the modal volume of CP-like modes in the gammadion metal cavities. The refractive indices of the undoped GaN and Al were obtained from the references by Palik as well as Peng and Piprek[1,2]. We setup three-dimensional models for the R- and L- gammadion metal cavities to compute electric fields of the modes. The gammadion was designed to have a linewidth, width, arm length, and height of 50, 305, 200, and 500 nm, respectively. The modal volume V for a mode in the GaN gammadion metal cavity, after some generalization for taking the material dispersion into account, is written as

$$V = V_{\mathrm{eff}}\left(\frac{n}{\lambda}\right)^3 = \frac{\int \mathrm{d}\mathbf{r}\epsilon_{\mathrm{r,g}}(\mathbf{r},\omega_{\mathrm{r}})|\mathbf{E}(\mathbf{r})|^2}{\max\left[\epsilon_{\mathrm{r,g}}(\mathbf{r},\omega_{\mathrm{r}})|\mathbf{E}(\mathbf{r})|^2\right]}\left(\frac{n}{\lambda}\right)^3, \tag{S.16}$$

where $\epsilon_{\mathrm{r,g}}(\mathbf{r},\omega) = \partial\mathrm{Re}[\omega\epsilon_{\mathrm{r}}(\mathbf{r},\omega)]/\partial\omega$is the relative group permittivity of the mode; ω_{r} is the resonant frequency at threshold; and $\mathbf{E}(\mathbf{r})$ is the corresponding electric field. This expression is numerically evaluated for a given mode to estimate the dimensionless effective volume $V_{\mathrm{eff}} \approx 2.56$.

References

1. Fa1. Peng, T. & Piprek, J. Refractive index of AlGaInN alloys. *Electron. Lett.* **32**, 2285 (1996).

2. *Handbook of optical constants of solids*. (Acad. Press, 1998).
